# Transforming Perspectives Through Virtual Exchange: A US-Egypt Partnership Part 1

**DOI:** 10.3389/fpubh.2022.877547

**Published:** 2022-05-17

**Authors:** Elizabeth A. Wood, Sarah L. Collins, Savanah Mueller, Nichole E. Stetten, Mona El-Shokry

**Affiliations:** ^1^Department of Environmental and Global Health, College of Public Health and Health Professions, University of Florida, Gainesville, FL, United States; ^2^College of Public Health and Health Professions, University of Florida, Gainesville, FL, United States; ^3^Department of Occupational Therapy, College of Public Health and Health Professions, University of Florida, Gainesville, FL, United States; ^4^Department of Medical Microbiology and Immunology, Faculty of Medicine, Ain Shams University, Cairo, Egypt

**Keywords:** collaborative online international learning (COIL), virtual exchange, internationalization, global public health, transformative learning theory (TLT)

## Abstract

With more classrooms within higher education mobilizing strategies for internationalization, collaborative online international learning (COIL), also referred to as virtual exchange, is an effective approach at offering intercultural competence through experiential learning. This strategy provides students who face barriers to international travel the opportunity to engage with students from other countries in meaningful ways, while enhancing and reinforcing course content. Grounded in the transformative learning theory, this study evaluates the effectiveness of a virtual exchange that was implemented within an undergraduate global public health course. The virtual exchange connected students from the University of Florida (within the US) with medical students in a microbiology course at Ain Shams University in Cairo, Egypt. Using adapted reflection prompts, we assessed the students' knowledge and learning before, during, and after the virtual exchange. This was coupled with a final paper to capture how personal backgrounds and experiences may contribute to their perception of the virtual exchange, as well as if they felt their global perspective had changed or shifted during the experience. Using directed content analysis for each of the measurements, two researchers coded the data independently to then present agreed upon salient themes to the larger group. Of the 28 randomly sampled students who participated in the virtual exchange, seven major themes emerged from the data: Connectedness; Openness; Acquisition of Knowledge and Skills; Communication; Cultural Identity; Anticipation of Options for New Roles, Relationships, and Actions; and Absence of Change. Through this evaluation it was clear there was a variance of different perspectives with many sampled students having diverse lived experiences that influenced their worldview prior to the virtual exchange. Despite course-related barriers, students acknowledged several facilitating factors that improved their intercultural competence and knowledge of course content. The integration of a virtual exchange within the classroom, with careful design and implementation, can provide a unique experience for students and an inclusive approach to learning.

## Introduction

With the rising belief that internationalization is critical to higher education institutions, differences in the ability to offer comprehensive services can affect the competitiveness of the institution (1). Internationalization is defined as the “institutional process that in some way internalizes the concept of openness to the world in all the activities and organizational aspects of the university, and it may even launch an internal transformation to prepare the university to act more directly on the international or global scene” (2, para 1). Through internationalizing curriculum in higher education institutions, students are more prepared to navigate a global economy and promote academic mobility (2, 3). The stewardship of internationalization within an institution is crucial in ensuring quality of education and advising, increasing academic mobility and collaborative scholarship, and building a culture focused toward inclusiveness and consistency of services across a campus. Internationalization may manifest in several ways within higher education, specifically through collaborations in teaching, study abroad programs, as well as the continued support of international students (4). The proliferation of instructors integrating an international or global dimension within their courses and/or curriculum has led to a more common and fluid use of collaborative online international learning (COIL), also referred to as virtual exchange.

## Collaborative Online International Learning

COIL is a contemporary pedagogical approach to offering international, online exchanges between students to foster intercultural competence, digital literacy, and global self-awareness predominantly among peers with different linguacultural backgrounds (5–7). Using internet-based tools and online platforms for sharing materials, COIL promotes meaningful exchanges with students who are in different geographical locations. It is reflective of the overarching pedagogical approach that informs experiences such as virtual exchange, which ultimately describes the mechanism in which university students receive immersive short-term cross-cultural experiences. Prior to COIL, many turned to other forms of cultural immersion such as study abroad programs (8). However, COIL offers a contemporary solution to having students integrate constructs of intercultural competence into their normal daily lives rather than being physically immersed in another geographic location (9). It has also been effective during the COVID-19 pandemic with travel restrictions. COIL can be integrated into a course on a small scale (e.g., international guest lecturer) or larger scale (4–5 week modules with synchronous/asynchronous exchanges). Through the integration of internationalization within curriculum, students are more likely to engender these facets of COIL as well as cultivate a synergy between coursework and international relationships that may be lacking in traditional faculty-led study abroad sojourns (10). Furthermore, cooperative learning, a subset of collaborative learning, within COIL bolsters active and team-based learning styles through meaningful activities where students work together to problem solve; thus, augmenting their interpersonal and social skills (4, 10–12). Moreover, through collaborative learning, students are able to procure more responsibility working together in groups in order to build their knowledge through active engagement (13).

Foreign language courses have dominated the virtual exchange arena for many years, however, with new technologies in the classroom coupled with the increased digital literacy due to COVID-19-induced classroom dynamic shifts, other disciplines are benefiting from international collaborations (14). Guided by Mezirow's transformative learning theory (15), this virtual exchange sought to explore what students' perceptions and attitudes were around cross-cultural collaborative online international learning as well as to determine how a COIL experience may impact students' global self-awareness, perspective-taking, and intercultural communication within a global public health setting (15). Likewise, whether cultural perspective can influence a student's social and learning behavior. Finally, researchers wanted to assess how the virtual exchange has impacted students' perceived gains in learning global public health. In order to develop more autonomous thinkers and capture the unconscious shift to consciousness within the transformative learning paradigm, this virtual exchange implemented self-reflection activities before, during, and after the global learning experience.

The goal of this study was to better understand students' attitudes and perceptions about cross-cultural collaborative online international learning, while also assessing how this COIL experience impacts students' global self-awareness, perspective-taking, and intercultural communication. Through implementing the stages of the Transformative Learning Theory, we hope to capture how a virtual exchange has impacted the students' perceived gains in learning global public health. For the purposes of this study, authors chose to use the term “virtual exchange” rather than the broader term COIL to capture the telecollaborative nature of this experience.

## Materials and Methods

### Virtual Exchange Preparation

This virtual exchange occurred with Ain Shams University (ASU) in Cairo, Egypt and the University of Florida (UF) in Gainesville, Florida in the United States (US). Faculty from ASU's undergraduate medical program and UF's undergraduate public health program collaborated to design and implement a 5-week module for students with a global health focus (Appendix A). Drs. Mona El-Shokry and Elizabeth Wood participated in a Virtual Exchange Training Program hosted by UF in Fall 2020 with the intention of implementing a virtual exchange program in March 2021. In total, there were 108 UF students total who participated in the virtual exchange through a global public health course and 32 ASU students who participated voluntarily in addition to their normal coursework. The discrepancy between groups was a consequence of more students being added to the UF global health course due to demand. Sixteen groups were randomly formed for the virtual exchange with six UF students and two ASU students per group to avoid having only one ASU student per group and having too large of groups due to the number of UF students participating. Despite having 16 smaller groups, students from both universities regularly conversed as a whole through the elearning Canvas shell that all students had access to.

### Course Pedagogy

Similar to a study abroad departure training, instructors hosted an online, synchronous Zoom meeting for ASU students and an onboarding training for UF students in February during the course semester. Onboarding included preparation for students with intercultural communication, reviewing diverging cultural norms and dimensions, as well as supplemental information on the respective country. A follow-up lecture was also provided midway through the virtual exchange to discuss culture shock, common stressors, and ways to navigate communicating with someone from another country.

**Figure 1:**
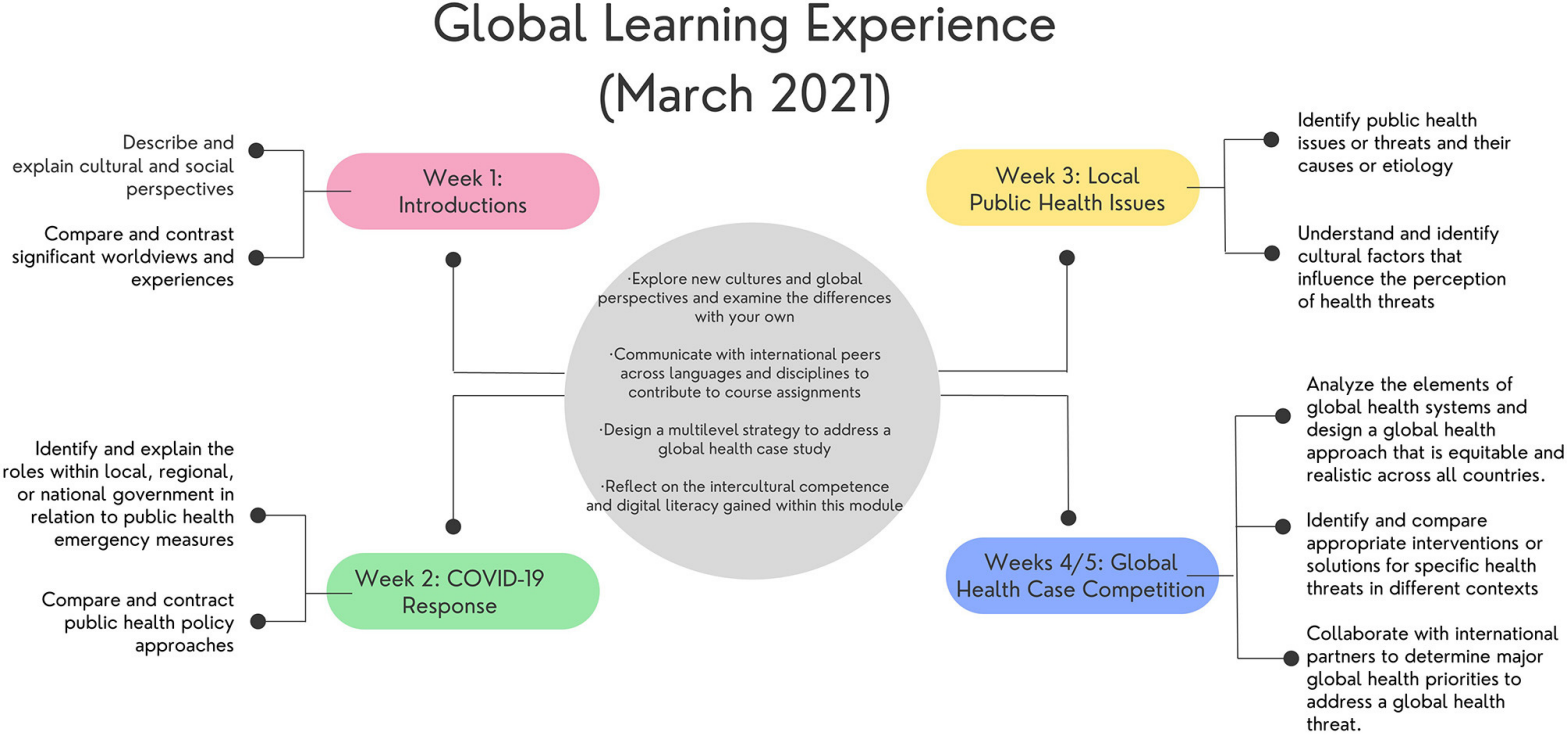
Global Learning Experience format.

The 5-week module was designed to have small group discussions within an elearning management system (Canvas) where students could exchange thoughts and ideas around weekly topics. However, students were not limited to Canvas and could communicate independently with each other through other modalities (e.g., Marco Polo, Zoom, WhatsApp, etc.). Figure 1 outlines the student learning objectives for each week, combining weeks four and five to accommodate a third wave of COVID-19 in Cairo at the time.

### Data Collection

UF students engaged in reflective activities throughout the virtual exchange program, including answering reflection questions provided by The State University of New York (SUNY) COIL Stevens Initiative Assessment (16). The assessment provides pre-, mid-, and post questions. Questions were adapted to reflect UF's global public health course objectives (Appendix B). The pre-, mid-, post responses were due during weeks zero, three, and five, respectively. In addition to the SUNY COIL assessment, an individual analysis paper (IAP) prompting students to reflect on the entirety of the virtual exchange experience was the final assessment (Appendix C).

Upon the conclusion of the semester, UF student responses to the outlined assessments were exported from Canvas and de-identified. Each student was provided a code to indicate their reported sex, race/ethnicity and a student number in order to match their assessments. Researchers organized students based on sex and found that only 11 out of the 108 UF students were male. To include all male participants, 2:1 matched sampling for each male's reported race/ethnicity was conducted^1^. Therefore, the researchers stratified the female students according to their reported race/ethnicity and randomly selected students using a random number generator to meet the 2:1 matching criterion. This sampling method yielded 35 students. Inclusion criteria required students to complete all four time points and to have answered each prompt in its entirety. The final sample was 28 students.

Data was organized according to each assessment item. For example, all student responses to pre-assessment question one were compiled into one document and labeled according to student ID. This was completed for pre-assessment questions two, three, and so forth for all assessment items. This data cleaning and management process is considered a useful strategy in establishing trustworthiness of a study (17). Trustworthiness depicts the quantitative equivalent, validity, with our efforts specifically aiming to enhance the study's credibility and the dependability or stability of the data (18). This study was approved by the University of Florida Institutional Review Board (IRB#: IRB202003293).

### Data Analysis

Student responses were analyzed using a combination of directed content analysis and inductive/open content analysis. The results from the directed content analysis will be the focus of this manuscript and the results of the inductive content analysis are expanded upon in Part B (19). The researchers used Mezirow's Transformative Learning Theory as the guiding theoretical framework for a directed content analysis (15), which utilizes deductive processes to code text to further expand on or validate an existing framework (20). Though there are traditionally 10 stages, the first stage, *disorienting dilemma*, was omitted from data analysis because the virtual exchange program itself is thought to be the disorienting dilemma. Further, the second stage was broken into two parts to illustrate the two-part reflection required to fulfill this theme. The researchers collaborated on developing appropriate operational definitions based on the existing language used for each learning stage. Themes and operational definitions used within the directed content analysis can be seen in Table 1. For codes that did not fit into the predetermined themes (Mezirow's 10 stages), inductive coding was used to identify repetitive codes. A more detailed account of inductive coding procedures can be found elsewhere (19).

**Table 1:** Codebook.

**Theme**	**Operational definition**
Self-examination with feelings of fear, anger, guilt or shame (1)	Internal reflection of held beliefs, attitudes, assumptions, biases, etc. [assumptions] and having an emotional response as they navigate through the reflection.
A critical assessment of assumptions–(2) Part A	An objective ponderance of initial reflection and clearly defining previous [assumptions].
A critical assessment of assumptions–(2) Part B	Within defining their previous [assumptions], they also recognize their aspiring [assumptions].
Recognition that one's discontent and the process of transformation are shared (3)	The acknowledgment that the individual is not isolated in experiencing dissonance and/or discomfort with previous [assumptions].
Exploration of options for new roles, relationships and action	Participant contemplation of options to integrate new [assumptions] via personal identity, interactions, and personal behaviors
Planning a course of action	Implicit or explicit presentation of how participants intend to employ new personal identity, interactions, and personal behaviors as related to refined [assumptions].
Acquiring knowledge and skills for implementing one's plans	Intentionally noted effort (or a clearly defined action item) to [actively] obtain knowledge from new information, as well as behavioral skills to implement one's course of action.
Provisional trying of new roles	Preliminary attempts to actively engage in new personal identity, interactions, and personal behaviors.
Building competence and self-confidence in new roles and relationships	Continued execution of new roles, thus explicit mention of expanding beyond the classroom context, practicing relevant behaviors on a routine basis, etc. which in turn, increases self-efficacy within new [assumptions].
A reintegration into one's life on the basis of conditions dictated by one's new perspective	An expression that the refined assumption is now perceived as habitual, routine, easy (i.e., without precontemplation) within one's everyday life.

Two coders independently performed directed content analysis, hand coding line-by-line. The researchers followed suggested qualitative analysis steps put forth by Mayring (21, 22) with minor adaptations to the outlined procedure. Independent analysis allowed each researcher to reach their own conclusions for data categorization within Mezirow's theoretical framework. Negotiations were performed after each data set was completed (Pre, Mid, Post, and IAP), rather than after working through 10–50% of the data, as suggested by Mayring (21, 22), to maintain consistency among data sets. During each negotiation, the researchers iteratively redefined the operational definitions based upon the findings and re-coded, line-by-line, until they reached 100% agreement, thereby increasing inter-coder reliability (23, 24). Following the completion of the IAP data analysis negotiation, the researchers revisited all data to ensure continued fit to the finalized theme operational definitions, as recommended by Hseih and Shannon (20).

A unique aspect of content analysis is the significance of a theme is partially determined by its prevalence (25). Upon completing all qualitative data analysis, the frequencies of each theme were quantified to determine each theme's overall prevalence. Themes were only counted once per student response per item, despite the frequency of presentation of the theme within each response. Furthermore, each student response may have multiple themes present.

## Results

The final sample for this study consisted of 28 randomly selected students from a total sample pool of 108 who completed the Global Learning Experience (GLE) within the UF undergraduate Global Public Health course. The majority of sample participants were female (68%). There was a wide distribution of racial and ethnic representation within the sample, including White, non-Hispanic (36%), White, Hispanic (25%), Black (25%), Asian (11%), and two or more races (3%). Final frequencies for each theme can be found in Table 2. Themes are presented below according to their position in Mezirow's Transformative Learning Theory (TLT).

**Table 2:** Frequency of themes.

**Themes**	**Categories (if applicable)**	**Data collection round**				
		**Pre-VE**	**Mid-VE**	**Post-VE**	**IAP**	**Total**
Self-examination with feelings of fear, anger, guilt, or shame		5	7	2	6	**20**
A critical assessment of assumptions		1	21	12	18	**52**
	A critical assessment of assumptions–Part A	12	13	10	10	**45**
	A critical assessment of assumptions – Part B	0	0	0	0	**0**
Recognition that one's discontent and the process of transformation are shared		1	0	1	0	**2**
Exploration of options for new roles, relationships, and action		0	3	11	7	**21**
Planning a course of action		0	0	12	3	**15**
Acquiring knowledge and skills for implementing one's plans		0	2	3	7	**12**
Provisional trying of new roles		0	1	7	6	**14**
Building competence and self-confidence in new roles and relationships		0	0	0	0	**0**
A reintegration into one's life on the basis of conditions dictated by one's new perspective		0	0	0	0	**0**

### Self-Examination With Feelings of Fear, Anger, Guilt, or Shame (1)

The first theme described when individuals express an internal reflection of their assumptions and have an emotional response as they navigate through the reflection. Though the title of this theme reflects negative emotions, these emotions were scarcely expressed. Among the few, one student simultaneously expressed gratitude and guilt when they state, “We are lucky that English is such a common language and I feel bad that we cannot converse in a language they are more comfortable with.”

Instead of the negative emotions, including fear, anger, guilt, or shame, students more commonly expressed feelings such as surprise or shock. One student noted, “What surprised me the most in this module was how some people can be unaware of cultural differences and would have to learn how to speak to others.” Another student expressed an emotional realization when engaging with the Egyptian partners, “I think that I honestly have been a little shocked by some of the differences in the issues we have discussed together.” One of the more interesting emotional reactions was one student's expression of relief. They note that though the GLE came as a culture shock, it made them “more relieved to see that our cultures had more similarities than the differences [they] had initially anticipated.” This may insinuate some preexisting anxiety in cross-cultural interactions and students' beliefs that they would not share any commonalities with their Egyptian counterparts. Identifying these positive emotional experiences provides a unique aspect of the TLT.

### A Critical Assessment of Assumptions (2)

Traditionally, the second theme is presented as a singular stage; however, to extract the nuanced phases of a critical assessment, two categories were developed: (2a) an objective baseline reflection and clearly defining previous assumptions; and (2b) within defining their previous assumptions, they also recognized their aspiring assumptions. If an individual presented both aspects within a single item response, they were classified under (2) “a critical assessment of assumptions.” In contrast, if an individual only presented a portion of the requirement to demonstrate a critical assessment, they were classified under the appropriate category. This theme, followed by category 2a, was the most prevalent TLT theme identified within student responses.

There were several accounts of comprehensive critical assessments (2). One student's comment can summarize a typical assessment, “It really helped me to not think so United States centric…” Students' ability to recognize their US-centric experiences assisted in their ability to assess their assumptions critically. One student explains, “I feel we are more likely to resort to thinking that countries outside the US are drastically different, but [the] truth is they have a lot more in common than we may perceive.” One student implicitly noted the relational discourses that are often reflected between American^2^ society and other countries, by stating, “I had the misconceived connotation that most countries…were technologically and industrially behind—prohibiting them from sharing a similar perspective to my own. However, after talking with my Egyptian peers and learning more…, this was a very generalized view of [the] entire population.” These examples demonstrate a transformation of thought where students actively refocused their assumptions that position American society parallel to international partners rather than at the center of conversation.

Interestingly, students who engaged in 2a also noted biases that are seemingly grounded in American norms without completely identifying a shift in perception and development of new assumptions. One student stated, “I think I tend to be biased and view life from an American-centered lens, but that is not true for the rest of the world,” while another echoed those sentiments by expressing, “Sometimes I feel as if we get stuck in a bubble here in America and believe everyone in the world functions at the same time we do, especially when a lot of us UF students do not communicate internationally on a daily basis.” One student expanded upon this idea and generalized beyond their own experiences. “My thought process was more in-line with a more outdated, inaccessible population. I believe Americans are under the impression that other countries, especially African countries, are struggling, and [its] citizens don't have access to many of the advancements we do.” These examples demonstrate a strong reflection of their assumptions but simply fail to explicitly state how their assumptions have changed. Predictably, no student engaged only in category 2b where they recognized their aspiring assumptions without first determining their preexisting assumptions.

### Recognition That One's Discontent and the Process of Transformation Are Shared (3)

This theme of the TLT only manifested twice in the data. This theme presents one's acknowledgment that they are not isolated in experiencing dissonance and/or discomfort with their previous assumptions. One student overtly stated, “As we talked about in class, we all had our previous thoughts about Egypt and Egyptians.” Though this does not explicitly state their preconceptions of Egypt or Egyptians, it highlighted that more than one individual had shared experiences. Interestingly, the other instance of this theme presented a collective “we” in overcoming assumptions, “I think that our assumptions about each other will be surprising to overcome as we learn how to work in international groups.” This presents a different facet of this theme. It demonstrated that a student's perception of a collective transformation is just as influential as an actual collective transformation. Though we don't know if other students openly shared their transformation process with this student, it is inferred that they believe their American peers were undergoing the same discontent and dissonance as them.

### Exploration of Options for New Roles, Relationships and Action (4)

This theme starts the beginning of a transition from a cognitive exploration to actionable behaviors in one's TLT experience. Specifically, this theme arises when individuals contemplate options to integrate their new assumptions based on the previous themes. Exploration of options ranged from the ways a student could immediately engage in new roles or relationships with their new assumptions to the delayed options such as career integration. For example, one student stated, “Also, [the GLE] has even given me a new view on the different people around me. For example, being at UF I may meet people from different cultural backgrounds and can now be more likely to be open to learning from them.” In contrast, another student explored how the GLE will influence their practice as a future physician, “I gained a new perspective on how another country may view Americans. I would be able to apply this perspective in the future with patients from another culture in making them more comfortable.” Though these examples range in implementation over the course of one's life, they provide specific examples where students can employ their new assumptions.

### Planning a Course of Action (5)

This theme represents an implicit or explicit presentation of how participants intended to employ their new assumptions. This theme predominately centers on how students plan to be an active agent in their learning, which is demonstrated through active statements to utilize their new assumptions. Some students discussed taking steps to validate their new skills, such as one who says, “In the future I would like to be able to visit another country and see for myself that I am able to use these newly gained cultural skills.” Others expressed their intention to increase their global awareness and competency by actively engaging in international news and events, “However, being in America it seems like most international news you hear is from China or maybe France or only if there is a natural disaster somewhere. I would now like to try to go out of my way to learn about current events internationally.”

### Acquiring Knowledge and Skills for Implementing One's Plans (6)

The acquisition of knowledge and skills is described as an intentional effort or action item to obtain new information to implement one's plan. One student said, “After reflecting on the Egyptian students' experiences, I contacted them privately with more questions.” This depicted how the Egyptian partners served as an invaluable resource due to their lived experiences providing unparalleled insight into cultural norms and experiences. Other students echoed this sentiment stating the experiences made them “want to listen and ask questions to [their] peers.” Students built upon the GLE relationships to obtain more detailed information or expand on a topic they had a personal interest in. For example, one student stated, “I want to explore other cultures' foods more! In our introductions, an international peer shared their favorite dish (chicken schwarma). I found a recipe and made it the next day- it was amazing!” Ultimately, this acquisition of knowledge and skills further the students' ability to implement their plan and simultaneously increase their cultural competence.

### Provisional Trying of New Roles (7)

When students reach this stage, they are working to codify their action plan. These include preliminary attempts to actively engage in new personal identity, interactions, and/or personal behaviors based on their new assumptions. One student plainly stated, “It also is great to undergo trial and error with working internationally which is a critical component in global health I am experiencing first hand.” Others described this experience by stating, “During the case study project, it was important for us to listen to the feedback [our Egyptian partner] had about what we wanted to try to implement in a university in Egypt. His feedback allowed us to ensure that what we were trying to do would be successful and allowed us to also tweak aspects that do not make sense in the context of an Egyptian university.” Finally, some students made statements that demonstrated their materialization of new assumptions. For example, one student stated, “I am glad that my experience with my Egyptian peers went well, and there are a few small takeaways that I was able to come up with, but these mainly relate to public health. An Egyptian person of a different economic status or race may have had completely different views or a different personality than our peers, so I believe small takeaways better than large generalities, which may lead to stereotypes.” Though not as explicit as the other examples, this depicts a cognitive shift that aims to avoid stereotypes and misconceptions, thus is an equally valuable example of implementation of new assumptions. It should be noted that not all students who presented provisionally trying new roles engaged in previous stages.

### Other Themes

Neither of the final two themes presented, “Building competence and self-confidence in new roles and relationships (8)” or “A reintegration into one's life on the basis of conditions dictated by one's new perspective (9)” were identified within the data. This may be due to the short nature of the GLE.

## Discussion

Through this iteration of a global public health course, the addition of a virtual exchange was clearly appreciated by the students, especially during a time when international travel was limited. The acquisition of knowledge through various forms of interactive, autonomous, scaffolded learning broadly improved intercultural competence and offered a synergistic way of teaching course content. In many cases interacting with their international peers offered a positive experience despite coping with frustrations such as a 6-h time difference and coordinating times to meet. Both positive and frustrating experiences are collectively captured within studies and reports that implemented a virtual exchange that yielded greater overall intercultural awareness (4, 16, 26). Despite never meeting face-to-face, students were still able to socially interact in meaningful ways through various virtual modalities. It was intentional that students were able to choose their own platform(s) for communication to ensure they were comfortable throughout the collaborative process. Both Driscoll in 1994 (27) and Graham and Misanchuk in 2004 (28) describe these interactive, group work activities as facilitating factors for the development of decision-making and problem-solving skills. Furthermore, Johnson and Johnson (29) state how within collaborative learning, students begin to appreciate heterogeneity, which can culminate into perspective-taking and feelings of assent.

The positive experiences that were captured through this study have been reinforced through previous literature, specifically around strengthening the application of course content (16, 30, 31). Findings demonstrate how through this virtual exchange, students can apply the skills they have learned in their future endeavors, be it within the workforce or higher education. Moreover, partnering with students who were not only international peers, but also from adjacent disciplines (medicine and microbiology), has been shown to improve veritable communication, comprehensive troubleshooting, and knowledge application (32, 33). Additionally, the findings related to student assumptions demonstrate how a substantial number of students reported that their international peers were quite similar to both themselves and practices within the US. This discovery heightens their nascent intercultural awareness and perspective-taking, and specifically addresses areas of ethnocentrism, such as asserting universal values (16, 34).

From a student learning perspective, the findings reveal that students generally either built upon pre-existing knowledge or created the foundation for new knowledge around course content and other cultures. While measuring cultural competence is required for many accrediting bodies in higher education, cultural humility more fully captures and represents the aim of this study. We recognize that this study captures change over time, however, we are also acutely aware that cultural humility and awareness is not finite and require lifelong learning and transformation (16, 35–37). Moreover, while cultural humility and awareness may have increased during this virtual exchange, it is likely to wax and wane as the students' lived experiences and perspectives unfold over time.

## Conclusion

During a time when international travel was untenable for most of the population, virtual exchange allowed for students and instructors to engage with international partners in the classroom. While virtual exchanges may not be a panacea for global interaction, the approach addresses several lacunae within the classroom around immersive dialogue, inclusive international experiences, and global self-awareness (16, 38, 39). The integration of virtual exchange, even in the most basic form, creates the opportunity for instructors to develop introspective, erudite students who are challenged into participating outside their comfort zone to foster greater self and cultural awareness.

## Limitations

Two limitations existed in this study including the data collection method and the time frame available. The data collection method followed a structured, written-response format, hindering further exploration of prominent inductive themes or ideas. This study may have benefited from data triangulation through in-person interviews after the conclusion of the exchange to support richer responses that may have proven beneficial in identifying the subtle changes along the transformative learning cycle. Semi-structured question interviews that support thematic analysis may have been a supplemental addition to the data collection.

Additionally, while the study spanned a 5-week time period, which was sufficient to demonstrate some momentous change along Mezirow's transformative learning theory, it may not have been sufficient to demonstrate the entire cycle. Had these students been followed up within 3–6 months after the conclusion of the virtual exchange, they might have demonstrated the entire cycle, which was not seen in our study. It was also impractical to assess a reintegration into one's life in such a short time span, as this encompasses activities outside the classroom. An opportunity for additional studies could be virtual exchanges performed over a longer period or incorporating an additional follow up period.

## Data Availability Statement

The datasets presented in this study can be found in online repositories. The names of the repository/repositories and accession number(s) can be found at: https://original-ufdc.uflib.ufl.edu/IR00011736/00001.

## Ethics Statement

The studies involving human participants were reviewed and approved by University of Florida Institutional Review Board. Written informed consent for participation was not required for this study in accordance with the national legislation and the institutional requirements. Written informed consent was not obtained from the individual(s) for the publication of any potentially identifiable images or data included in this article.

## Author Contributions

EW contributed to preliminary discussions regarding research design and corresponding methodologies, collected data, wrote the introduction, part of the methods, discussion, and conclusion sections of this manuscript. SC contributed to preliminary discussions regarding research design and corresponding methodologies, conducted data analysis procedures, wrote the pedagogical theory, methods, and results sections of this manuscript. SM conducted data analysis procedures, wrote the results, and limitations sections of this manuscript. NS contributed to preliminary discussions regarding research design and corresponding methodologies, as well as training and supervising data analysis procedures. ME-S contributed to the initial design and implementation of the study. All authors engaged in editing the manuscript.

## Conflict of Interest

The authors declare that the research was conducted in the absence of any commercial or financial relationships that could be construed as a potential conflict of interest.

## Publisher's Note

All claims expressed in this article are solely those of the authors and do not necessarily represent those of their affiliated organizations, or those of the publisher, the editors and the reviewers. Any product that may be evaluated in this article, or claim that may be made by its manufacturer, is not guaranteed or endorsed by the publisher.

## Footnotes

^1^There were no reported “two or more races” males, but researchers still randomly selected two females from this ethnic/racial group.

^2^America/n in this context refers to a person from the United States.
