# Supplementary material for: Transforming Perspectives Through Virtual Exchange: A US-Egypt Partnership Part 1
Source: Front Public Health. 2022 May 17;10:877547. doi: 10.3389/fpubh.2022.877547 (PMC9152246; doi:10.3389/fpubh.2022.877547)
Supplement: Supplementary file 1 [file Table_1.DOCX]

**University of Florida**

**College of Public Health & Health Professions Syllabus**

**PHC 3440: Global Public Health (3 credits)**

**Global Learning Experience (GLE)**

**GLE Overview**

This Global Learning Experience (GLE) is broken up into two sections, Virtual Exchange and Individual Reflection. The purpose of this module is to grant students the ability to learn and engage with people, rather than media, books, or lectures. Through international student engagement, students will experience the complexity of multiple perspectives, questioning of their own assumptions, and intercultural awareness as part of building toward being a civic and globally engaged learner.

**GLE Objectives**

- Explore new cultures and global perspectives and examine the differences with your own
- Communicate with international peers across languages and disciplines to contribute to course assignments
- Design a multilevel strategy to address a global health case study
- Reflect on the intercultural competence and digital literacy gained within this module

**GLE Activities**Students will have trainings prior to the GLE in cultural humility and country-specific content (e.g. Egyptian culture). Students are also expected to review the Student Code of Conduct in regards to engaging with students in a professional and respectful manner. While the below activities are required, all students are welcome to engage respectfully with their international peer outside of class.

*Week 1: Introduce yourself*Within this assignment, students are expected to introduce themselves within a designated mode of communication (e.g. GLE Canvas shell). This can be done through text or video. Additionally, students should share with their international counterparts what a day looks like for students at your university. Respond to an international peer’s post.

*Objectives:*

- Describe and explain cultural and social perspectives
- Compare and contrast significant worldviews and experiences

*Week 2: COVID-19 Response*Within this post, discuss with your group how your country addressed its COVID-19 response. You may also discuss how your state or region addressed the response as well. What public health measures were taken and when? What could have been done better within your country? Globally? Respond to an international peer’s post indicating the similarities and differences within the two countries.

*Objectives:*

- Identify and explain the roles within local, regional, or national government in relation to public health emergency measures
- Compare and contract public health policy approaches

*Week 3: Local Public Health Issues*Provide an example of a public health issue of concern within your community, this can be local, regional, or national. Why is it a major public health issue? Describe what measures are being taken to address this issue and evaluate whether these measures are effective. Post a response to an international peer with potential public health strategies that may address their issue.

*Objectives:*

- Identify public health issues or threats and their causes or etiology
- Understand and identify cultural factors that influence the perception of health threats

*Week 4: Global Health Case Study*At the end of week 3 all students will be given a mock global health case study. Each small group will have until the end of Week 4 to design a strategy to address the case study specifically. Students will present their strategy in a presentation and will be judged by faculty from both universities. Closing ceremonies will take place at the end of Week 4 where students from both universities exchange what they have learned about the other, and the impact it has had on their learning and understanding of global health content.

*Objectives:*

- Analyze the elements of global health systems and design a global health approach that is equitable and realistic across all countries.
- Identify and compare appropriate interventions or solutions for specific health threats in different contexts
- Collaborate with international partners to determine major global health priorities to address a global health threat.

**GLE Individual Analysis Paper**Students are required to submit a final, culminating reflection paper on the GLE experience by the due date. Within this reflection students will address areas such as how their international peer impacted their learning experience, what key changes in perceptions of the world occurred, how your personal cultural background affected your understanding of this experience, among other areas of reflection and input.
