# Supplementary material for: Transforming Perspectives Through Virtual Exchange: A US-Egypt Partnership Part 1
Source: Front Public Health. 2022 May 17;10:877547. doi: 10.3389/fpubh.2022.877547 (PMC9152246; doi:10.3389/fpubh.2022.877547)
Supplement: Supplementary file 2 [file Table_2.DOCX]

Appendix B. Adapted SUNY COIL Pre-, Mid-, Post- Assessments (14)

| **Pre-GLE Prompts**   1. How do you think your interaction with students from another country might impact what you learn in this course? (30-50 words) 2. How do you think the way you see and understand the world might change by connecting with students in another country? (30-50 words) 3. How would you describe your cultural background? (30-50 words) 4. What do you want from this virtual exchange experience? (30-50 words)   **Mid-GLE Prompts**   1. Provide two or more observations about your interactions with your partner(s) as you work with them in the online environment. (30-50 words) 2. Describe how your course has been impacted by connecting with a class from another country. (30-50 words) 3. Now that you have connected with students from another country, how are your views of your partner(s) culture changing? Please provide examples. (30-50 words) 4. How do you think that your cultural background impacts the way in which you interpret course content and interact with your partner(s)? Please provide examples. (50-100 words) 5. What surprises you about the virtual exchange experience? (30-50 words)   **Post-GLE Prompts**   1. What was the most important thing you learned from this collaborative course? (30-50 words) 2. Please describe how doing this course collaboratively with international partner(s) impacted your learning experience? (30-50 words) 3. Given your online interactions with students from another country, describe any key changes that occurred in how you view the world? (30-50 words) 4. How did your cultural background influence your understanding of this course material and shape your interaction with your partner(s)? (50-100 words) 5. Was there any aspect of this virtual exchange -enhanced course that was stressful in any way? If so, please describe this challenge and what you learned from it? (30-50 words) 6. What do you want to explore further as a result of this connection to students from another country? (50-100 words) 7. Was there anything you like would like this experience to improve upon in the future? (unlimited) |
| --- |
