# Supplementary material for: Transforming Perspectives Through Virtual Exchange: A US-Egypt Partnership Part 1
Source: Front Public Health. 2022 May 17;10:877547. doi: 10.3389/fpubh.2022.877547 (PMC9152246; doi:10.3389/fpubh.2022.877547)
Supplement: Supplementary file 3 [file Table_3.DOCX]

Appendix C. Individual Analysis Paper Assignment

"A mind that is stretched by a new experience can never go back to its old dimensions.” *– Oliver Wendell Holmes, Jr.*

For this paper, describe your experiences during the Virtual Exchange and what you have (or have not) learned by answering the following questions:

- Describe your own culture, traditions, and/or background and explain how these individual or family traits have shaped your views of how you approached working collaboratively with your international peers.
- What elements (Canvas page, social networking, etc.) did you find most effective at learning and interacting with your international peer and why. What barriers or challenges did you and your group have to overcome (language, technology, etc.)?
- How has your global perspective changed or shifted (if at all) during this learning experience? In other words, how has this activity broadened your understanding of how people in other countries address global health threats?

Optional:

- If there is any additional thoughtful information you would like to provide in this essay, please label it "Optional" and include it at the end of the above prompts.

Please use APA formatting for references and citations, double-spaced 2-3 pages, 12 font, Times New Roman.

Please review the Global Learning Rubric below to understand what will receive full credit.

**GLOBAL LEARNING VALUE RUBRIC**

| GLOBAL LEARNING VALUE RUBRIC | | |
| --- | --- | --- |
| **Criteria** | **Ratings** | **Pts** |
| This criterion is linked to a Learning Outcome  Global Self-Awareness: in the context of global learning, the continuum through which students develop a mature, integrated identity with a systemic understanding of the interrelationships among the self and those around them | \| **20 to >12.0 pts**  **Full Marks**  Effectively addresses significant issues in the natural and human world based on articulating one’s identity in a global context. \| **12 to >9.0 pts**  **Milestone**  Evaluates the global impact of one’s own and others’ specific local actions on the natural and human world. \| **9 to >6.0 pts**  **Milestone**  Analyzes ways that human actions influence the natural and human world. \| **6 to >0 pts**  **Benchmark**  Identifies some connections between an individual’s personal decision-making and certain local and global issues. \| \| --- \| --- \| --- \| --- \| | 20 pts |
| This criterion is linked to a Learning Outcome  Perspective Taking: the ability to engage and learn from perspectives and experiences different from one’s own and to understand how one’s place in the world both informs and limits one’s knowledge. The goal is to develop the capacity to understand the interrelationships between multiple perspectives, such as personal, social, cultural, disciplinary, environmental, local, and global. | \| **20 to >12.0 pts**  **Full Marks**  Evaluates and applies diverse perspectives to complex subjects within natural and human systems in the face of multiple and even conflicting positions (i.e. cultural, disciplinary, and ethical.) \| **12 to >9.0 pts**  **Milestone**  Synthesizes other perspectives (such as cultural, disciplinary, and ethical) when investigating subjects within natural and human systems. \| **9 to >6.0 pts**  **Milestone**  Identifies and explains multiple perspectives (such as cultural, disciplinary, and ethical) when exploring subjects within natural and human systems. \| **6 to >0 pts**  **Benchmark**  Identifies multiple perspectives while maintaining a value preference for own positioning (such as cultural, disciplinary, and ethical). \| \| --- \| --- \| --- \| --- \| | 20 pts |
| This criterion is linked to a Learning Outcome  Knowledge Application: An ability to apply knowledge and skills gained through higher learning to real-life problem-solving both alone and with others. | \| **20 to >12.0 pts**  **Full Marks**  Applies knowledge and skills to implement sophisticated, appropriate, and workable solutions to address complex global problems using interdisciplinary perspectives independently or with others. \| **12 to >9.0 pts**  **Milestone**  Plans and evaluates more complex solutions to global challenges that are appropriate to their contexts using multiple disciplinary perspectives (such as cultural, historical, and scientific). \| **9 to >6.0 pts**  **Milestone**  Formulates practical yet elementary solutions to global challenges that use at least two disciplinary perspectives (such as cultural, historical, and scientific). \| **6 to >0 pts**  **Benchmark**  Defines global challenges in basic ways, including a limited number of perspectives and solutions. \| \| --- \| --- \| --- \| --- \| | 20 pts |
| This criterion is linked to a Learning Outcome Communication  Navigating the various forms of communication (e.g. Canvas, WhatsApp, Zoom, etc.) to communicate with international peers. | \| **20 to >15.0 pts**  **Full Marks**  This ability to speak to the various technological media that were used throughout the project. Include failures and successes as necessary. \| **15 to >9.0 pts**  **Milestone**  Included a brief description of technologies used. \| **9 to >0 pts**  **No Marks**  Not describing the technologies used \| \| --- \| --- \| --- \| | 20 pts |
| This criterion is linked to a Learning Outcome Formatting  Follows APA format, 2 - 3 pages, double spaced, 12 in font, Times New Roman. Spelling and grammar. | \| **20 to >0.0 pts**  **Full Marks** \| **0 pts**  **No Marks** \| \| --- \| --- \| | 20 pts |
| Total Points: 100 | | |

*Adapted from [AACU Global Learning Value Rubric](https://www.aacu.org/value/rubrics/global-learning) (40)
